# Differentially expressed miR-3680-5p is associated with parathyroid hormone regulation in peritoneal dialysis patients

**DOI:** 10.1371/journal.pone.0170535

**Published:** 2017-02-02

**Authors:** Sohyun Jeong, Jung Mi Oh, Kook-Hwan Oh, In-Wha Kim

**Affiliations:** 1 College of Pharmacy and Research Institute of Pharmaceutical Sciences, Seoul National University, Seoul, Republic of Korea; 2 Division of Nephrology, Department of Internal Medicine, Seoul National University Hospital, Seoul, Republic of Korea; Hospital Universitario de la Princesa, SPAIN

## Abstract

Mineral and bone disorder (MBD) is observed universally in patients with chronic kidney disease (CKD). Detrimental MBD-related skeletal changes include increased prevalence of fracture, cardiovascular disease, and mortality. MicroRNAs (miRNAs) have been identified as useful biomarkers in various diseases, and the aim of this study was to identify miRNAs associated with parathyroid hormone level in peritoneal dialysis (PD) patients. Fifty-two PD patients were enrolled and grouped by their intact parathyroid hormone (iPTH) level; 11 patients had low iPTH (<150 pg/mL) and 41 patients had high iPTH (≥150 pg/mL). Total RNA was extracted from whole blood samples. Total RNA from 15 patients (7 and 8 patients in the low and high iPTH groups, respectively) underwent miRNA microarray analysis, and three differentially upregulated (>2-fold change) miRNAs previously associated with human disease were selected for real-time quantitative PCR (qPCR) analysis. Interaction analyses between miRNAs and genes were performed by using TargetScan and the KEGG pathway database. Microarray results revealed 165 miRNAs were differentially expressed between patients with high iPTH levels and low iPTH levels. Of those miRNAs, 81 were upregulated and 84 were downregulated in patients with high iPTH levels. Expression levels of miR-1299, miR-3680-5p, and miR-548b-5p (previously associated with human disease) in 52 patients were analyzed by using qPCR. MiR-3680-5p was differentially expressed in low and high iPTH patients (*P* < 0.05). The predicted target genes of miR-3680-5p were *USP6*, *USP32*, *USP46*, and *DLT*, which are involved in the ubiquitin proteolysis pathway. This pathway has roles in PTH and parathyroid hormone related protein degradation and proteolysis. The mechanisms involved in the associations among low PTH, adynamic bone disease, miR-3680-5p, and the target genes should be explored further in order to elucidate their roles in CKD-MBD development.

## Introduction

Chronic kidney disease (CKD) is a public health condition, affecting 13% to25% of the worldwide population. [1, 2]. Mineral and bone disorder (MBD) is a common systemic complication of CKD that can begin as early as CKD stage 3 in the course of failing kidney function. A damaged kidney cannot effectively excrete phosphorus, nor can it produce the active metabolite 1,25(OH)_2_D (calcitriol), thereby leading to secondary hyperparathyroidism (SHPT). The disrupted mineral and endocrine functions in CKD are vital factors in the regulation of bone metabolism. As a result, altered bone remodeling and loss of bone volume are observed extensively in dialytic CKD and in the majority of CKD stages 3–5 patients. [3–5]. In addition to SHPT, the prevalence of adynamic bone disease (ABD) is not negligible in CKD patients and is observed in 10% to 71% of patients in CKD stage 5D [6–9]. These bone remodeling problems result in a higher prevalence of hip fracture in CKD patients than in the healthy population [10–16], furthermore, hip fracture in CKD stages 3–5 patients with is associated with a mortality rate twice that in non-dialysis patients with a hip fracture [12, 17]. Additionally, cardiovascular disease is associated with 70% of all deaths in patients with CKD. Coronary artery calcification is one of the primary causes of cardiovascular-related death in patients on dialysis as it can lead to cardiac ischemia and sudden death [18–20].

Increased awareness of the detrimental effects of CKD-MBD has resulted in extensive research focused on finding mechanisms and diagnostic bone markers related to CKD-MBD development. The main bone markers studied to date are parathyroid hormone (PTH), alkaline phosphatase, tartrate-resistant acid phosphatase, bone collagen-derived peptides, and non-collagenous bone proteins. Of those markers, PTH is the most reliable reference marker of bone disease, with high levels (>600 pg/mL, 9 folds the upper limit of normal) indicating increased bone resorption and turnover (*e*.*g*., SHPT) and low levels (<150 pg/mL,2 folds the upper limit of normal) indicating decreased bone resorption and turnover (*e*.*g*., ABD). Moreover, both high and low levels of PTH are potentially harmful to the cardiovascular system as both high and low bone turnover rates are associated with increased vascular calcification and mortality [21, 22].

The correlation of serum PTH level and bone histomorphometric parameters of renal osteodystrophy (ROD) can replace diagnostic bone biopsy at a moderate degree of accuracy [23, 24]. That can be accomplished in part because variable amounts of PTH fragments may have specific effects that are antagonistic to that of the intact molecule, and because normal and low bone turnover rates occur over a wide range of PTH levels (*i*.*e*., 2- to 9-fold the upper normal limit of the PTH assay) [25]. In 2009, a new PTH threshold 2–9 times that of the upper normal limit [26] compared to the conventional PTH level of 150–300 pg/mL [27] was suggested by the Kidney Disease Improving Global Outcomes (KDIGO) for CKD stage 5 patients. Due to the differences in PTH assay methods and reference ranges, the use of PTH level as the primary tool for defining and monitoring bone turnover changes in CKD patients has not fully elucidated the complex bone disease process. Thus, there is a demand to identify additional makers associated with changes in PTH level and bone disease.

MicroRNAs (miRNAs) are small non-coding RNAs involving in post-transcriptional regulation of gene expression. Identification of miRNA function can indicate novel targets for genetic regulation across a wide spectrum of biological processes of cell differentiation, organogenesis, and development [28–30]. Disregulated miRNA expression has a crucial role in the pathogenesis of genetic and complicated disorders, as well as in cancers, inflammatory diseases, and cardiovascular disease [31, 32]. Differential miRNA expression is associated with disease status, suggesting potential roles of miRNAs as diagnostic, prognostic, and predictive markers [33]. It has been suggested that miRNA profiles can be significantly altered in parathyroid adenoma, and parathyroid miRNAs are essential for the development of SHPT, regulation of PTH; moreover, miRNAs can be used as bone disease biomarkers [34, 35]. However, there is limited information available describing the relationships among miRNAs, PTH regulation, and bone disease. The aim of the present study was to identify the role of miRNAs in end-stage renal disease (ESRD) patients with CKD-MBD and to elucidate possible links between miRNAs and the traditional bone turnover serum marker PTH. The results will assist in describing the mechanisms associated with high or low bone turnover disease in ESRD patients.

## Materials and methods

### Patients and protocol

This prospective study was carried out between July 2015 and February 2016 in a nephrology department of a tertiary hospital in Seoul, Korea. The study was approved by the hospital’s ethics committee (IRB: H-1507-098-689). According to the approved IRB procedure, two written informed. consents for study participation and genetic testing of blood samples were obtained from all study subjects. The documents of written consents and consents activity were recorded and secured. Inclusion criteria were: age 18 years or older and required to undergo chronic maintenance peritoneal dialysis (PD). Exclusion criteria included systemic active infection, severe liver disease, active malignancy, parathyroidectomy history, pregnancy, and immunosuppressant treatment. Patients indicated for intact PTH (iPTH) testing (secondary generation, normal range 10–65 pg/mL) to detect mineral disorders and that satisfied the selection criteria were asked to enroll in the study. Fifty-two patients fulfilled the selection criteria and agreed to enroll in the study. Routine laboratory blood examinations for ESRD patients were conducted for all study participants. Blood samples were collected at the same day as clinical evaluation.

### MiRNA expression analysis

#### Blood sample preparation

A 2.5 mL sample of whole blood from each patient was drawn directly to a PAXGene Blood miRNA Kit (PreAnalytiX, Qiagen BD, Manchester, UK), held for 2 h att ambient temperature (18–25°C), and then stored at −80°C in a freezer prior to performing miRNA expression analysis. Total RNA quality and quantity were assessed by using an Agilent 2100 Bioanalyzer system (Agilent Technologies, Santa Clara, CA, USA).

#### Microarray analysis

Analysis of human miRNA expression was initiated by using a miRCURY LNA microRNA Array kit (Exiqon, Vedbaek, Denmark). Processed microarray slides were then scanned with an Agilent G2565CA Microarray Scanner System (Agilent Technologies). Scanned images were imported by Agilent Feature Extraction software version 10.7.3.1 (Agilent Technologies), and fluorescence intensities of each image were quantified by applying the modified manufacturer’s protocol.

#### qPCR analysis with a Taqman method

Among the differentially expressed miRNAs identified by microarray analysis, three miRNAs reported to be related to human disease were selected for qPCR analysis. The expression levels of miR-548b-5p, miR-3680-5p, and miR-1299 were measured by using the appropriate TaqMan MicroRNA Assay (ABI assay IDs 002408, 465029_mat, and 241065_mat, respectively; Foster City, CA, USA). A 384-well high-throughput analysis was performed by using the ABI Prism 7900 Sequence Detection System (PE Applied Biosystems, Foster City, CA, USA). The reverse transcriptase reactions contained a 10 ng RNA sample, 50 nM stem-loop RT primer, 1 × RT buffer, and 0.25 mM of each dNTP, 3.33 U/μL MultiScribe reverse transcriptase, and 0.25 U/μL RNase Inhibitor (Applied Biosystems). The synthesized cDNA was amplified, and the thermal cycling conditions included initial denaturation at 95°C for 10 min followed by a reaction cycle (95°C for 30 s and 60°C for 1 min) that was repeated 40 times. The quantitative fluorescence data were analyzed by using sequence detection software (SDS version 2.2, PE Applied Biosystems). The cycle number at which the amplification plot crossed the threshold was calculated, and the threshold cycle (Ct) value was normalized to U6-snRNA (ABI assay ID 001973). Relative quantification of miRNA expression was calculated by using the 2^−ΔΔCt^ method.

### MiRNA target prediction and pathway analysis

The target genes associated with the three selected differentially expressed miRNAs were determined by searching the TargetScan 6.2 database (http://www.targetscan.org/vert_71/). The target prediction runs were performed with a context percentile of 95% and a conserved method. To locate miRNA targets within various biological networks, the characterization of target gene list was based on the functional annotation terms contained within the KEGG pathway database [36]. Gene to miRNA associations were classified according to their level of statistical significance. Fisher's exact test was used to determine the over representation of specific biochemical pathways statistically. The probability was computed for each annotation term based on a hypergeometric distribution. We estimated the likelihood that the selected target genes actually belonged to a specific annotation term by applying the model presented by Tavazoie *et al*. (1999) [37]. Pathways with a Benjamini-Hochberg adjusted *P*-value < 0.01 were deemed significant.

### Statistical analysis

The required sample size for microarray was calculated assuming log scale gene expression was under normal distribution. The power to detect 2-fold difference of expression between two groups was 80%, significance level was p value <0.05. According to this calculation, the sample size per group was approximately 8 per group [38] Since there was no known established sample size calculation for qPCR, all the PD patients who satisfied the inclusion criteria during the research period were asked to participate. The significance of the differences in miRNA expression levels derived from microarray results between the low iPTH (<150 pg/mL) and high iPTH (≥150 pg/mL) groups were determined by using Mann-Whitney *U* tests. The *P*-values were corrected by using the Benjamini-Hochberg procedure. The correlations between miRNA expression levels and iPTH levels were explored using both Pearson correlation and Spearman correlation analyses. Differences in qPCR-derived expression levels between the two groups were analyzed by applying Mann-Whitney *U* tests. Conditional logistic regressions were performed to determine the independent risk factors associated with a low iPTH level. Independent variables included in the multivariate analysis were those with significant univariate analysis results. Statistical analyses were conducted by using SPSS software (version 22, IBM SPSS, Chicago, IL, USA), and all tests of significance were based on a two-sided 0.05 level.

## Results

### Patient characteristics

Fifty-two PD patients met the selection criteria and agreed to participate in the study. The participant’s median (range) age was 51.5 (23–72) years, and 23 patients were male (44.23%). The patients’ causes of ESRD included glomerular nephritis (n = 24), hypertension (n = 7), diabetes (n = 9), and others (n = 12, which included autosomal dominant polycystic kidney disease, vesicoureteral reflux disease, renal anomalies, etc.). The median (range) PD duration was 61.5 (16.0–93.5) months. Renal Kt/V_urea_ was calculated using data from 24-hour collection of urine. Peritoneal Kt/V_urea_ and creatinine clearance (Ccr) were calculated by performance of 24-hour collection of dialysate effluent. Total Kt/Vurea was measured by the sum of renal and peritoneal Kt/V_urea_. Median (range) biochemical laboratory results were: iPTH 304.5 (67.0–1366) pg/mL, Ca 9.1 (7.5–10.3) mg/dL, P 5.2 (2.6–9.3) mg/dL, alkaline phosphatase 87 (36.0–225.0) IU/L, and hemoglobin 10.3 (7.3–13.6) g/dL. The iPTH-based distribution of the participants was <150 pg/mL iPTH (n = 11), 150–300 pg/mL iPTH (n = 14), 300–600 pg/mL iPTH (n = 20), and >600 pg/mL iPTH (n = 7).

Total twenty-nine (55.8%) of the participants had bone problems; 19 patients had bone dual energy X-ray absorptiometry (DEXA) confirmed osteopenia, 7 patients had DEXA confirmed osteoporosis, 2 patients had fracture history, and 1 patient had renal osteodystrophy. Baseline demographic and biochemical characteristics of patients with low iPTH (<150 pg/mL) and high iPTH (≥150 pg/mL) were compared (Table 1), and only hemoglobin and phosphorous levels were significantly different between the two iPTH groups.

**Table 1:** Demographic and baseline biochemical parameter characteristics of study participants.

Characteristics	Patients (N = 52)		*P* value
	1. Low iPTH 2. (<150 pg/mL) 3. (N = 11)	1. high iPTH 2. (≥150 pg/mL) 3. (N = 41)	
Male sex, n (%)	6 (54.5)	17 (41.5)	0.442
Age, years, median (range)	54 (24–72)	50 (23–69)	0.346
BMI, kg/cm^2^, median (range)	20.89 (17.5–28.1)	22.45 (16.57–29.73)	0.308
Cause of CKD, N(%)			0.785
DM	1 (9.1)	8 (19.5)	
HTN	2 (18.2)	5 (12.2)	
GN	6 (54.5)	18 (43.9)	
others	2 (18.2)	10 (24.4)	
Dialysis information, median(range)			
Kt/V_urea_	1.9 (1.52–3.03)	1.89 (1.50–3.69)	0.875
duration, month	17.06 (1.13–238.83)	63.77 (4.1–225.03)	0.554
Biochemical level, median (range)			
corrected calcium (mg/dL)	9.54 (8.42–9.84)	9.24 (8.0–10.42)	0.996
Phosphorus (mg/dL)	4 (2.9–8.4)	5.7 (3.9–9.3)	**0.022**
ALP (IU/L)	69.5 (45–121)	92 (36–225)	0.208
Hb (g/dL)	11.2 (7.6–13.6)	10.2 (7.3–11.9)	**0.004**
Albumin (g/dL)	3.7 (3.1–4.1)	3.8 (3–5)	0.542
Serum creatinine (mg/dL)	6.21 (10.77–21.22)	12.73 (3.95–25)	0.485
Na (mmol/L)	138 (131–143)	138 (126–144)	0.860
K (mmol/L)	4.3 (3.6–6.1)	4.8 (3.2–7.2)	0.131

**Abbreviation**s: BMI, body mass index; CKD, chronic kidney disease; DM, diabetes mellitus; HTN, hypertension; GN, Glomerulonephritis; K_t_/V: (K_urea_ × T_d_)/V_urea_ (K_urea_ is the effective (delivered) dialyzer urea clearance integrated over the entire dialysis, T_d_ is the time measured from beginning to end of dialysis, and V_urea_ is the patient’s volume of urea distribution; iPTH, intact parathyroid hormone; ALP, alkaline phosphatase; Hb, hemoglobin. *Significant results were marked in bold

### Association of miRNAs expression and PTH level change

#### Microarray analysis

At initial enrollment, 15 patients were selected for miRNA expression microarray analysis. Given that an iPTH level of 150 pg/mL is indicated as the lower limit of normal bone turnover in dialysis patients, 8 patients with iPTH ≥ 150 pg/mL and 7 patients with iPTH < 150 pg/mL had their miRNA profiles compared. All the microarray data was deposited in GEO (GSE90991: http://www.ncbi.nlm.nih.gov/geo/query/acc.cgi?token=fparzqkgqugqexi&acc=GSE90991). The identities of the miRNAs detected by microarray analysis were confirmed by accessing the mirBase (http://www.mirbase.org), microRNA.org (http://www.microrna.org/microrna/home.do), and mirDB (http://mirdb.org/miRDB) databases.

**Fig 1:**
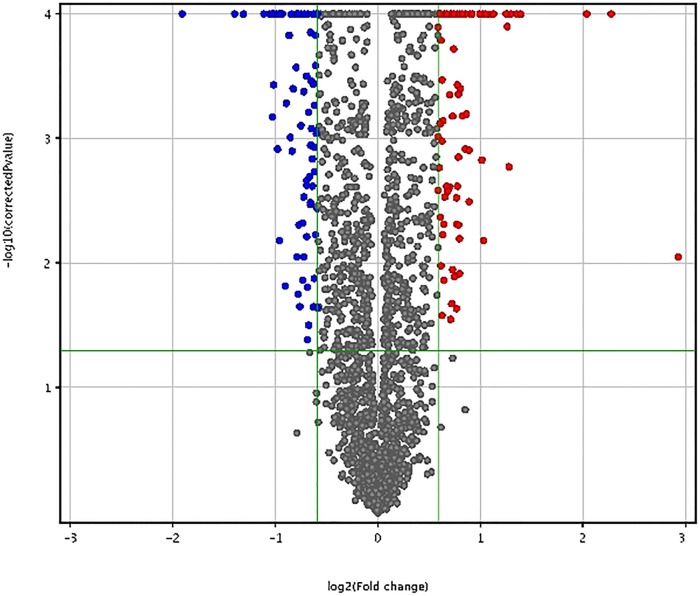
Volcano plot for differentially expressed miRNAs in patients with low (<150 pg/mL) and high (≥150 pg/mL) iPTH levels.

A mean of 1918 miRNAs were detected in the blood samples obtained from the 15 assessed PD patients. The differential expression results, after *P*-value adjusted for multiple testing by the Benjamini-Hochberg correction, demonstrated that the expressions of 165 miRNAs were significantly different (adjusted *P*-value <0.05) between the low and high iPTH patients. Of these, 81 miRNAs were upregulated and had a mean (range) fold increase of 1.91 (1.50–7.59), whereas 84 miRNAs were downregulated and had a mean (range) fold decrease of 1.75 (1.50–3.74) (Fig 1).

**Fig 2:**
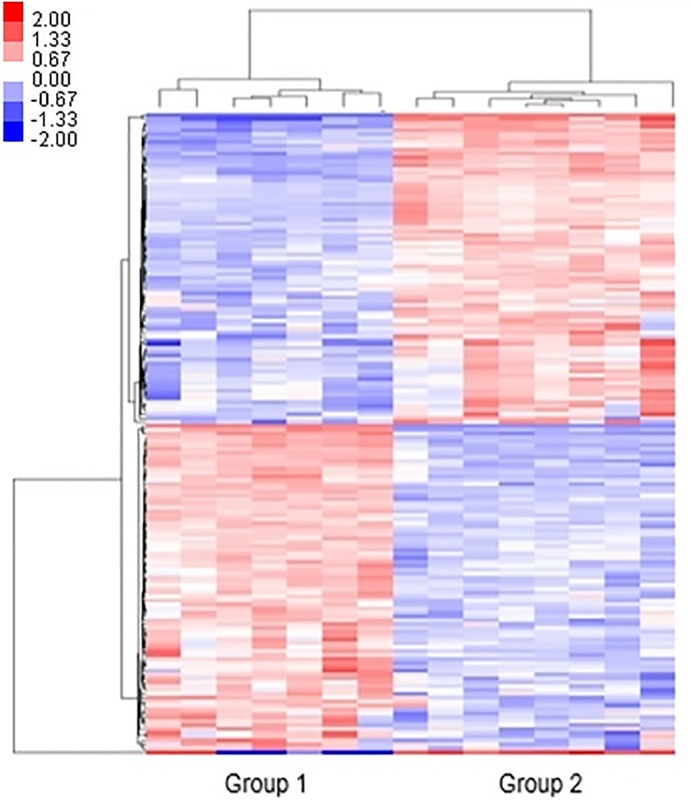
Heat map illustrating miRNAs profiles in patients with low (<150 pg/mL) and high (≥150 pg/mL) iPTH levels. The log_2_ values were calculated for each sample by normalizing to the count number of reads alone. The heat map analysis was performed by using Cluster 3.0 with the Euclidean distance algorithm and average linkage (*P*_adj_ <0.05 and log_2_ fold change >2). Group 1: iPTH < 150 pg/mL, Group 2: iPTH ≥ 150 pg/mL

The upregulated and downregulated miRNAs associated with low and high iPTH levels in the 15 assessed PD patients are listed in S1 Table and S2 Table. Fig 2 shows a heat map representation of the differentially expressed miRNAs in patients with low and high iPTH levels.

#### Real-Time PCR analysis

**Fig 3:**
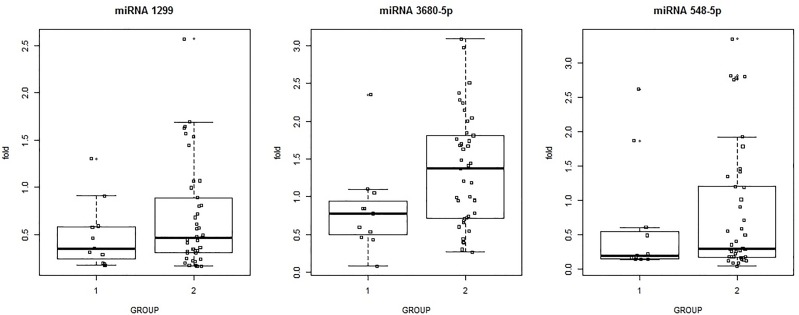
Association between iPTH level and upregulation of expression of three miRNAs in 52 PD patients.

According to the results of the microarray analysis, three of the most differentially expressed miRNAs with previously documented functional characteristics related to human diseases were selected for further analysis. That investigation involved performing qPCR of all 52 study participants. Among the differentially expressed candidate miRNAs, three (miR-548b-5p, miR-3680-5p, and miR-1299) were markedly upregulated (fold changes 4.85, 4.09, and 2.56, respectively). Upregulation of MiR-3680-5p was significantly different (*P* < 0.05) between the PD patients with low and high iPTH levels, whereas upregulations of miR-1299 and miR-548b-5p were not significantly different between the low and high iPTH groups (Fig 3).

Pearson and Spearman Correlation were performed between iPTH level and 3-miRNAs, and phosphate and 3-miRNAs, to find out any association among the variables. The result presented that only miR-3680-5P was associated with iPTH level (*P* = 0.045), with *r* = 0.279.

Univariate logistic regression results showed that phosphorous, hemoglobin, and miRNA-3680-5p levels were significantly associated with iPTH level. Accordingly, phosphorous, hemoglobin, and miRNA-3680-5p data were included in a multivariate logistic regression analysis. The multivariate logistic regression results showed that phosphorous was not significantly associated with iPTH level (*P* = 0.120), but both hemoglobin and miRNA-3680-5p were significantly associated with iPTH level (*P* < 0.05) (Table 2).

**Table 2:** Results of multivariate logistic regression analysis of clinical data and miRNAs in patients with low (<150 pg/mL) and high (≥150 pg/mL) iPTH levels.

Variables	Exp (B)	95% CI	*P* value
Hemoglobulin	0.336	0.142–0.796	0.013
miR-3680-5p	6.235	1.184–32.829	0.031

### MiRNA target prediction and pathway analysis

Target gene prediction was performed by using TargetScan with the 95% context percentile and conserved method (S3 Table). Among the 16 predicted genes listed, the most cited genes (*USP6*, *USP32*, *USP46* and *DTL*) were annotated to the ubiquitin-dependent protein catabolic process and protein deubiquitination genome elements, according to the KEGG pathway database (Table 3).

**Table 3:** Pathways and genes predicted to be associated with miR-3680-5p.

GO ID	GO Term	Genes	FDR corrected *P* value
0006511	Ubiquitin-dependent protein catabolic process	*USP6*,*DTL*,*USP32*,*USP46*	0.005
0016579	Protein deubiquitination	*USP6*, *USP32*, *USP46*	0.030

## Discussion

In this study, we identified miRNAs associated with PTH level which can reference ABD in ESRD patients. Through microarray analysis, 165 miRNAs were identified to be associated with lower limit of normal PTH level in PD patients. Three of those miRNAs (miR-548b-5p, miR-3680-5p, and miR-1299) had been previously reported to be related to human diseases such as cancer [39], tuberculosis [40], and rheumatic heart disease [41]. Those three were selected for further analysis by using qPCR.

The results of the analyses indicate that miR-3680-5p can be associated with ABD, because the expression of miR-3680-5p is significantly lower in PD patients having a iPTH level less than 150 pg/mL than it is in PD patients with a iPTH level greater than 150 pg/mL. The results of the target gene analysis revealed that miR-3680-5p was annotated to *USP 6*, *USP 32*, *USP 46*, and *DLT;* genes associated with ubiquitin-dependent proteolysis and deubiquitination.

The appropriate PTH level suggested in the 2009 KDIGO guidelines for ESRD patients was based on bone biopsy results for CKD-MBD patients. The data reviewed for those guidelines showed that the highest frequent form of renal osteodystrophy (ROD) in PD patients was ABD (50%), followed by mild bone disease (20%), osteitis fibrosa (18%), mixed bone disease (5%), and osteomalacia (5%). Only 2% of those PD patients had normal bone histology. [26]. From these results, PD should be an important risk factor for ABD etiology. Moreover, compared to other forms of ROD, the prevalence of ABD has been increasing and may be the most frequent type of bone lesion, particularly among diabetic patients. [7, 9, 42–46]. The presence of ABD in ESRD patients may be suspected by assessing the results of biochemical tests such as the detection of a low PTH level. For example, a PTH level less than twice the upper limit of normal (classically, a low PTH level in dialysis population is below 150 pg/mL according to the 2003 KDOQI guideline) may indicate the presence of ABD [25, 42, 47].

ABD is characterized by markedly lowered osteoblast and osteoclast numbers, bone formation rate, and activation frequency (a marker of bone turnover). The main risk factors for ABD are age, uremia, excessive PTH suppression from vitamin D analogues, calcium overload, PD, and diabetes mellitus [48, 49]. Moreover, ABD is related with a decreased ability to repair bone microdamage [50] and accumulation of such damage may cause increased fracture risk [11, 51]. Histomorphometric analysis of a tetracycline double-labeled bone biopsy is the current gold standard for ROD diagnosis. However, the invasive course of that procedure, along with its high cost and overall complexity, limits the efficacy of using the bone biopsy approach,[52] thus the requirement for developing non-invasive bone disease markers is increasing.

In this study, miR-3680-5p was associated with the lower limit of PTH (150 pg/mL) and those results suggested that miR-3680-5p could provide a research target in the study of non-invasive biomarkers and ABD etiology. Moreover, miR-3680-5p was associated with the *USP6*, *USP32*, *USP46*, and *DTL* genes, which have roles in the ubiquitin (deubiquitin) proteolysis system. ABD is characterized by bone resistance to bone-anabolic PTH functions, assumed as via downregulation of the PTH and parathyroid hormone related protein (PTHrP) receptors on osteoblasts [48, 53]. It has been reported that PTHrP plays a role in normal cell proliferation and differentiation, including a critical role in skeletogenesis [54–56]. The *PTHLH* and *PTHR1* genes, major regulators of mineral and bone metabolism, are subject to degradation and proteolysis by the actions of the ubiquitin-dependent pathway [48, 53]. Although the associated mechanism has not been fully described, miR-3680-5p can have a role in downregulation of ubiquitin-dependent pathway genes, which in turn can result in downregulation of PTH/PTHrP degradation via ubiquitin-dependent proteolysis. Such actions affect bone remodeling and resorption.

We could not determine the exact mechanism involved in the association between miR-3680-5p and the PTHrP coding genes; however, we were able to observe an indirect mechanism involving miR-3680-5p and the ubiquitin-dependent proteolysis pathway genes. Thus, further research to clarify the association between miR-3680-5p and PTH/PTHrP, and the mechanisms related to that association, is necessary. Regardless, the preliminary indications provided by the results of this study suggest that miR-3680-5p should be a research target for further study into the etiology of CKD-MBD. Moreover, our results provided insights into the search for non-invasive biomarkers that may be useful in identifying high risk ABD patients.

## Supporting information

S1 Table Differentially upregulated miRNAs in patients with high (≥150 pg/mL) and low (<150 pg/mL) iPTH levels. (DOCX)

S2 Table Differentially downregulated miRNAs in patients with high (≥150 pg/mL) and low (<150 pg/mL) iPTH levels. (DOCX)

S3 Table Predicted target genes determined by using TargetScan 6.2 with a 95% context percentile and conserved method. (DOCX)
