# Supplementary material for: Differentially expressed miR-3680-5p is associated with parathyroid hormone regulation in peritoneal dialysis patients
Source: PLoS One. 2017 Feb 2;12(2):e0170535. doi: 10.1371/journal.pone.0170535 (PMC5289431; doi:10.1371/journal.pone.0170535)
Supplement: S1 Table — (DOCX) [file pone.0170535.s001.docx]

Supplementary Table S1. Differentially upregulated miRNAs in patients with high (≥150 pg/mL) and low (<150 pg/mL) iPTH levels

| miRNA name | mirBase_ID | log FC ratio | P value | Benjamini-Hochberg HDR | Chr |
| --- | --- | --- | --- | --- | --- |
| hsa-miR-548b-5p | [MIMAT0004798](http://www.mirbase.org/cgi-bin/mirna_entry.pl?acc=MIMAT0004798) | 2.278 | 9.27E-21 | 8.89E-18 | chr6 |
| hsa-miR-3680-5p | [MIMAT0018106](http://www.mirbase.org/cgi-bin/mirna_entry.pl?acc=MIMAT0018106) | 2.087 | 2.18E-08 | 8.21E-07 | chr16 |
| hsa-miR-1299 | [MIMAT0005887](http://www.mirbase.org/cgi-bin/mirna_entry.pl?acc=MIMAT0005887) | 1.389 | 3.24E-09 | 2.32E-07 | chr9 |
| hsa-miR-34c-3p | [MIMAT0004677](http://www.mirbase.org/cgi-bin/mirna_entry.pl?acc=MIMAT0004677) | 1.376 | 1.02E-08 | 4.53E-07 | chr11 |
| hsa-miR-34c-5p | [MIMAT0000686](http://www.mirbase.org/cgi-bin/mirna_entry.pl?acc=MIMAT0000686) | 1.326 | 9.11E-09 | 4.18E-07 | chr11 |
| hsa-miR-5004-3p | [MIMAT0021028](http://www.mirbase.org/cgi-bin/mirna_entry.pl?acc=MIMAT0021028) | 1.309 | 1.39E-04 | 7.23E-04 | chr6 |
| hsa-miR-218-2-3p | [MIMAT0004566](http://www.mirbase.org/cgi-bin/mirna_entry.pl?acc=MIMAT0004566) | 1.272 | 3.72E-06 | 4.03E-05 | chr5 |
| hsa-miR-1539 | [MIMAT0007401](http://www.mirbase.org/cgi-bin/mirna_entry.pl?acc=MIMAT0007401) | 1.268 | 2.68E-09 | 2.06E-07 | chr18 |
| hsa-miR-337-5p | [MIMAT0004695](http://www.mirbase.org/cgi-bin/mirna_entry.pl?acc=MIMAT0004695) | 1.192 | 2.79E-08 | 9.74E-07 | chr14 |
| hsa-miR-548h-3p | [MIMAT0022723](http://www.mirbase.org/cgi-bin/mirna_entry.pl?acc=MIMAT0022723) | 1.173 | 1.44E-07 | 3.27E-06 | chr8 |
