# Supplementary material for: Differentially expressed miR-3680-5p is associated with parathyroid hormone regulation in peritoneal dialysis patients
Source: PLoS One. 2017 Feb 2;12(2):e0170535. doi: 10.1371/journal.pone.0170535 (PMC5289431; doi:10.1371/journal.pone.0170535)
Supplement: S2 Table — (DOCX) [file pone.0170535.s002.docx]

| **Supplementary Table S2. Differentially downregulated miRNAs in patients high (≥150 pg/mL) and low (<150 pg/mL) iPTH levels** | | | | | |
| --- | --- | --- | --- | --- | --- |
| miRNA name | mirBase_ID | Log fold ratio | P value | Benjamini-Hochberg FDR | Chr |
| hsa-miR-191-3p | [MIMAT0001618](http://www.mirbase.org/cgi-bin/mirna_entry.pl?acc=MIMAT0001618) | -1.917 | 2.12E-21 | 4.06E-18 | chr3 |
| hsa-miR-1207-3p | [MIMAT0005872](http://www.mirbase.org/cgi-bin/mirna_entry.pl?acc=MIMAT0005872) | -1.372 | 2.36E-08 | 8.55E-07 | chr8 |
| hsa-miR-4278 | [MIMAT0016910](http://www.mirbase.org/cgi-bin/mirna_entry.pl?acc=MIMAT0016910) | -1.327 | 4.50E-09 | 2.81E-07 | chr5 |
| hsa-miR-5010-3p | [MIMAT0021044](http://www.mirbase.org/cgi-bin/mirna_entry.pl?acc=MIMAT0021044) | -1.138 | 8.39E-09 | 4.18E-07 | chr17 |
| hsa-miR-548ah-5p | [MIMAT0018972](http://www.mirbase.org/cgi-bin/mirna_entry.pl?acc=MIMAT0018972) | -1.093 | 4.43E-05 | 2.93E-04 | chr4 |
| hsa-miR-500a-5p | [MIMAT0004773](http://www.mirbase.org/cgi-bin/mirna_entry.pl?acc=MIMAT0004773) | -1.050 | 8.81E-04 | 0.00315905 | chrX |
| hsa-miR-4800-5p | [MIMAT0019978](http://www.mirbase.org/cgi-bin/mirna_entry.pl?acc=MIMAT0019978) | -1.047 | 7.15E-08 | 1.96E-06 | chr4 |
| hsa-miR-1276 | [MIMAT0005930](http://www.mirbase.org/cgi-bin/mirna_entry.pl?acc=MIMAT0005930) | -1.045 | 2.16E-12 | 1.04E-09 | chr15 |
| hsa-miR-4312 | [MIMAT0016864](http://www.mirbase.org/cgi-bin/mirna_entry.pl?acc=MIMAT0016864) | -1.042 | 3.59E-09 | 2.37E-07 | chr15 |
| hsa-miR-548ap-5p | [MIMAT0021037](http://www.mirbase.org/cgi-bin/mirna_entry.pl?acc=MIMAT0021037) | -1.025 | 8.99E-05 | 4.93E-04 | chr15 |
| hsa-miR-424-3p | [MIMAT0004749](http://www.mirbase.org/cgi-bin/mirna_entry.pl?acc=MIMAT0004749) | -1.006 | 1.64E-10 | 2.83E-08 | chrX |
| hsa-miR-3660 | [MIMAT0018081](http://www.mirbase.org/cgi-bin/mirna_entry.pl?acc=MIMAT0018081) | -1.002 | 2.16E-07 | 4.10E-06 | chr5 |
