# Supplementary material for: Differentially expressed miR-3680-5p is associated with parathyroid hormone regulation in peritoneal dialysis patients
Source: PLoS One. 2017 Feb 2;12(2):e0170535. doi: 10.1371/journal.pone.0170535 (PMC5289431; doi:10.1371/journal.pone.0170535)
Supplement: S3 Table — (DOCX) [file pone.0170535.s003.docx]

Supplementary Table 3. Predicted target genes performed by TargetScan 6.2 with a 95% context percentile and conserved method

| Symbol | Description | Chromosome | *P* value |
| --- | --- | --- | --- |
| *RBPMS* | RNA binding protein with multiple splicing | 8 | 0.0154 |
| *SCAF8* | SR-related CTD associated factor 8 | 6 | 0.0198 |
| *PGM2L1* | phosphoglucomutase 2-like 1 | 11 | 0.0176 |
| *IGSF3* | immunoglobulin superfamily member 3 | 1 | 0.0371 |
| *SAMD12* | sterile alpha motif domain containing 12 | 8 | 0.0371 |
| *DTL* | denticleless E3 ubiquitin protein ligase homolog | 1 | 0.0198 |
| *LRRN3* | leucine rich repeat neuronal 3 | 7 | 0.0044 |
| *USP46* | ubiquitin specific peptidase 46 | 4 | 0.0066 |
| *EFCAB1* | EF-hand calcium binding domain 1 | 8 | 0.0044 |
| *TXNDC15* | thioredoxin domain containing 15 | 5 | 0.0154 |
| *ARMC2* | armadillo repeat containing 2 | 6 | 0.0044 |
| *USP32* | ubiquitin specific peptidase 32 | 17 | 0.0219 |
| *FUT10* | fucosyltransferase 10 (alpha (1,3) fucosyltransferase) | 8 | 0.0066 |
| *LYRM7* | LYR motif containing 7 | 5 | 0.0066 |
| *USP6* | ubiquitin specific peptidase 6 | 17 | 0.0241 |
| *CD163* | CD163 molecule | 12 | 0.0154 |
